# A radiomics based approach using adrenal gland and periadrenal fat CT images to allocate COVID-19 health care resources fairly

**DOI:** 10.1186/s12880-023-01145-9

**Published:** 2023-11-10

**Authors:** Mudan Zhang, Xuntao Yin, Wuchao Li, Yan Zha, Xianchun Zeng, Xiaoyong Zhang, Jingjing Cui, Zhong Xue, Rongpin Wang, Chen Liu

**Affiliations:** 1 Department of Medical Imaging, International Exemplary Cooperation Base of Precision Imaging for Diagnosis and Treatment, NHC Key Laboratory of Pulmonary Immune-related Diseases, Guizhou Provincial People’s Hospital, No. 83 Zhongshan East Road, Nan Ming District, 550002 Guiyang, Guiyang, Guizhou Province China; 2 School Of Medicine, Guizhou University, 550000 Guiyang, Guizhou province China; 3 Department of Radiology, Guangzhou Women and Children’s Medical Center, Guangzhou Medical University, Guangzhou, China; 4 Department of Nephrology, Guizhou Provincial People’s Hospital, 550002 Guiyang, Guizhou province China; 5 Shanghai United Imaging Intelligence, Co., Ltd, 201807 Shanghai, China; 6 Department of Radiology, Southwest Hospital, Third Military Medical University (Army Medical University), 400038 Chongqing, China

**Keywords:** Adrenal gland, Periadrenal fat, Auto-segmentation, COVID-19, Radiomics

## Abstract

**Background:**

The value of radiomics features from the adrenal gland and periadrenal fat CT images for predicting disease progression in patients with COVID-19 has not been studied extensively. We assess the value of radiomics features from the adrenal gland and periadrenal fat CT images in predicting COVID-19 disease exacerbation.

**Methods:**

A total of 1,245 patients (685 moderate and 560 severe patients) were enrolled in a retrospective study. We proposed a 3D V-net to segment adrenal glands in onset CT images automatically, and periadrenal fat was obtained using inflation operation around the adrenal gland. Next, we built a clinical model (CM), three radiomics models (adrenal gland model [AM], periadrenal fat model [PM], and fusion of adrenal gland and periadrenal fat model [FM]), and radiomics nomogram (RN) after radiomics features extracted.

**Results:**

The auto-segmentation framework yielded a dice value 0.79 in the training set. CM, AM, PM, FM, and RN obtained AUCs of 0.717, 0.716, 0.736, 0.760, and 0.833 in the validation set. FM and RN had better predictive efficacy than CM (*P* < 0.0001) in the training set. RN showed that there was no significant difference in the validation set (mean absolute error [MAE] = 0.04) and test set (MAE = 0.075) between predictive and actual results. Decision curve analysis showed that if the threshold probability was between 0.4 and 0.8 in the validation set or between 0.3 and 0.7 in the test set, it could gain more net benefits using RN than FM and CM.

**Conclusions:**

Radiomics features extracted from the adrenal gland and periadrenal fat CT images are related to disease exacerbation in patients with COVID-19.

**Supplementary Information:**

The online version contains supplementary material available at 10.1186/s12880-023-01145-9.

## Background

The coronavirus disease 2019 (COVID-19) has caused serious public health problems. Although a diagnosis and treatment protocol suitable for the national situation has been enacted in each country, the consensus is to separate moderate and severe patients who need special care. Predicting early disease progression and accurately identifying which patients are at risk of developing severe or critical disease is not only conducive to early disease control. However, it will also effectively allocate healthcare resources globally.

Because it was unclear that the polymerase chain reaction (PCR) technique [1], clinical symptoms, and laboratory tests were correlated with progression in patients with COVID-19 [2], researchers started exploring predicting the disease prognosis of COVID-19 combining artificial intelligence (AI) technology and CT images. Zhang et al. [3] constructed a comprehensive system and proved that the combination of chest CT and AI technology is a potential tool to predict disease progression in patients with COVID-19. Most studies have focused on the relationship between features from chest CT images and disease progression and ignored the endocrine system, which plays an essential role in disease progression [4–6]. However, studies showing whether the prognosis of patients with COVID-19 can be affected by direct damage or indirect change of adrenal glands or periadrenal fat are lacking.

The entire system and the organs are closely related to the immune-inflammatory state (7–8). Moreover, the adrenal gland can be changed not only by receiving a direct attack from SARS-CoV-2 through angiotensin-converting enzyme-2 (ACE2) [9] but also by adapting to physiological needs and pathological conditions because it has an astonishing regenerative capacity to adjust to unique micro-environments [10]. Therefore, we hypothesized that adrenal gland changes could occur due to its adaptive mechanism and SARS-CoV-2 invasion. The periadrenal fat was bound to be affected as the closest tissue around the adrenal gland. Fundamental changes can be detected by extracting radiomics features from chest CT images using AI technology [11]. Manual segmentation is the first and critical step in successfully constructing a radiomics model, but it is time-consuming and error-prone because of repeated labor. Combining the auto-segmentation framework based on AI technology with manual revision improvesefficiency and quality (12–13).

We, therefore, constructed an auto-segmentation framework and developed a radiomics nomogram (RN) to predict progression in patients with COVID-19 by integrating radiomics features from adrenal glands and periadrenal fat CT images with clinical indicators and to determine that radiomics features extracted from the adrenal gland and periadrenal fat CT images were related to possibility of deterioration in patients with COVID-19.

## Methods

### Data resources and image grouping

**Fig. 1:**
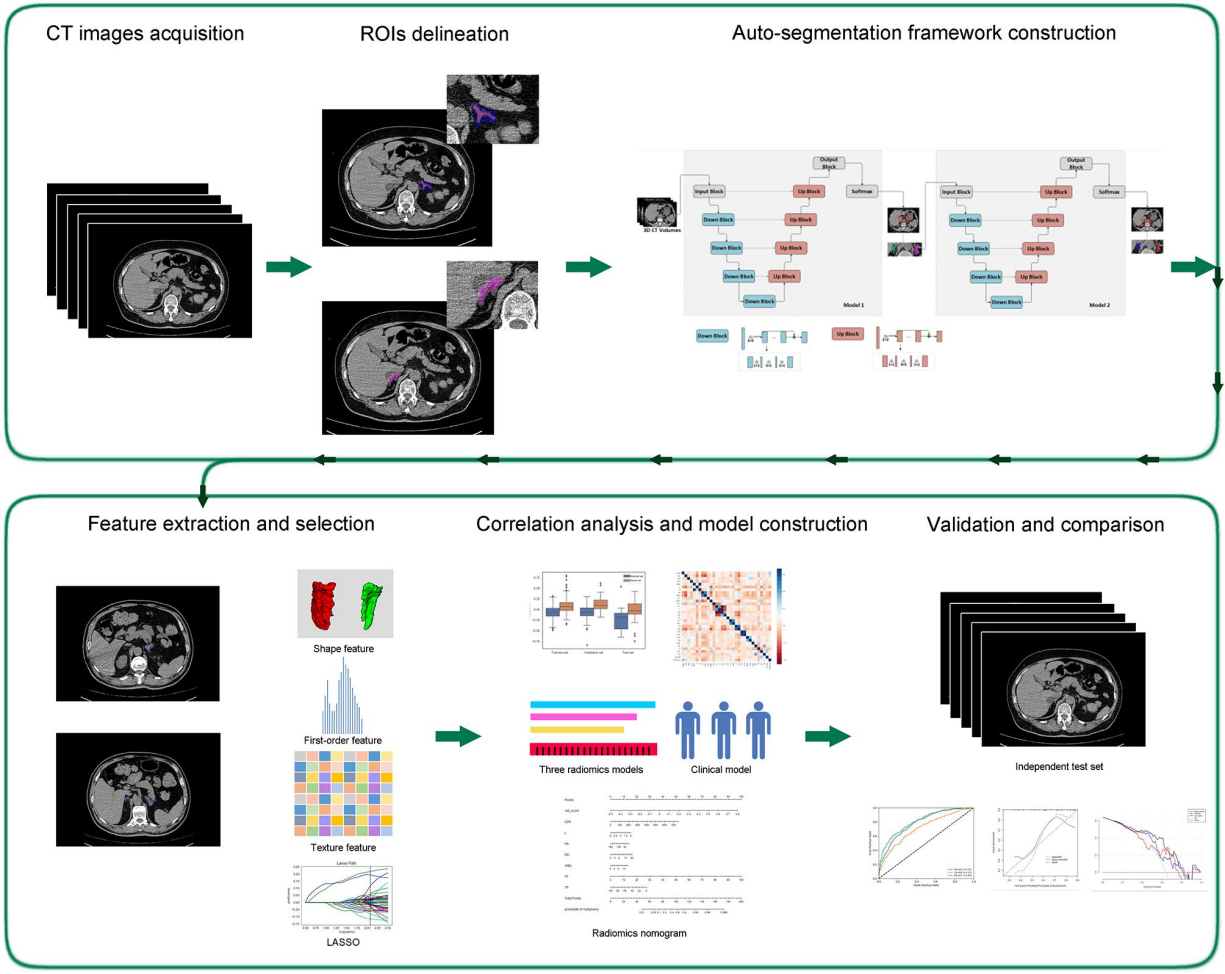
The workflow of the study

CT images and clinical data were retrospectively and consecutively collected between January 1 and April 30, 2020, from two hospitals: Huoshenshan Hospital (HSH) (n = 1,209) and Maternal and Child Health Hospital Optical Valley Branch Hospital of Hubei Province (MCH) (n = 36), China. Diagnosis and clinical classification of patients with COVID-19 were confirmed according to Diagnosis and Treatment Protocol for Novel Coronavirus Pneumonia (Trial Version 8). 30 clinical indicators were collected including age, sex, lnterleukin 6 (IL-6) levels, white blood cell count (WBC), lymphocyte count (L), neutrophil count (N), hemoglobin (HB ), red blood cell (RBC), blood platelet count (PLT), C-reactive protein (CRP), procalcitonin (PCT), prothrombin time (PT), thrombin time (TT), fibrinogen (FIB), D dimer (DD), blood sugar (BS), alanine transaminase (ALT), glutamic oxaloacetic transaminase (AST), total protein (TP), albumin (ALB), total bilirubin (TBil), direct bilirubin (D-Bil), blood urea nitrogen (BUN), creatinine (Cre), B-type natriuretic peptide (BNP), myoglobin (Mb), troponin (Tn), lactate dehydrogenase (LDH), creatine kinase (CK) and creatine kinase-MB (CK-MB). Moreover, the missing value, more than 20% of the total data, were excluded. If there were incomplete clinical or blood examination data, we used the median of the variable distribution to fix it. Patients of age ≤ 14 years (n = 2), low quality or unreadable chest CT images, and lesion or changes in adrenal glands CT images ( verified by two experienced radiologists) (n = 209) were excluded. All images were non-enhanced chest CT images and reconstructed at a slice thickness of 1.00 mm. Details of CT characteristics are listed in Supplementary Table 1. Patients with adrenal lesions were excluded after being evaluated by two radiologists with more than ten years of experience. We chose chest CT images scanned within four days of the first diagnosis as the onset image. If the CT scan was done more than once, we chose the one closer to the admission date. Supplementary Fig. 1 demonstrates the inclusion and exclusion criteria. Figure 1 shows the workflow of our study.

CT images of 1,209 patients from Huoshenshan Hospital were used to construct the prediction models divided into the training set (967/1209, 80%) and the validation set (242/1209, 20%) randomly. We constructed five models: the adrenal gland radiomics model (AM), periadrenal fat radiomics model (PM), a fusion of the adrenal gland and periadrenal fat radiomics model (FM), clinical model (CM) and radiomics nomogram (RN). A total of 36 Patients (13 moderate and 23 severe patients from Maternal and Child Health Hospital Optical Valley Branch Hospital) were included as an independent test set.

### Auto-segmentation framework and ROIs delineation

**Fig. 2:**
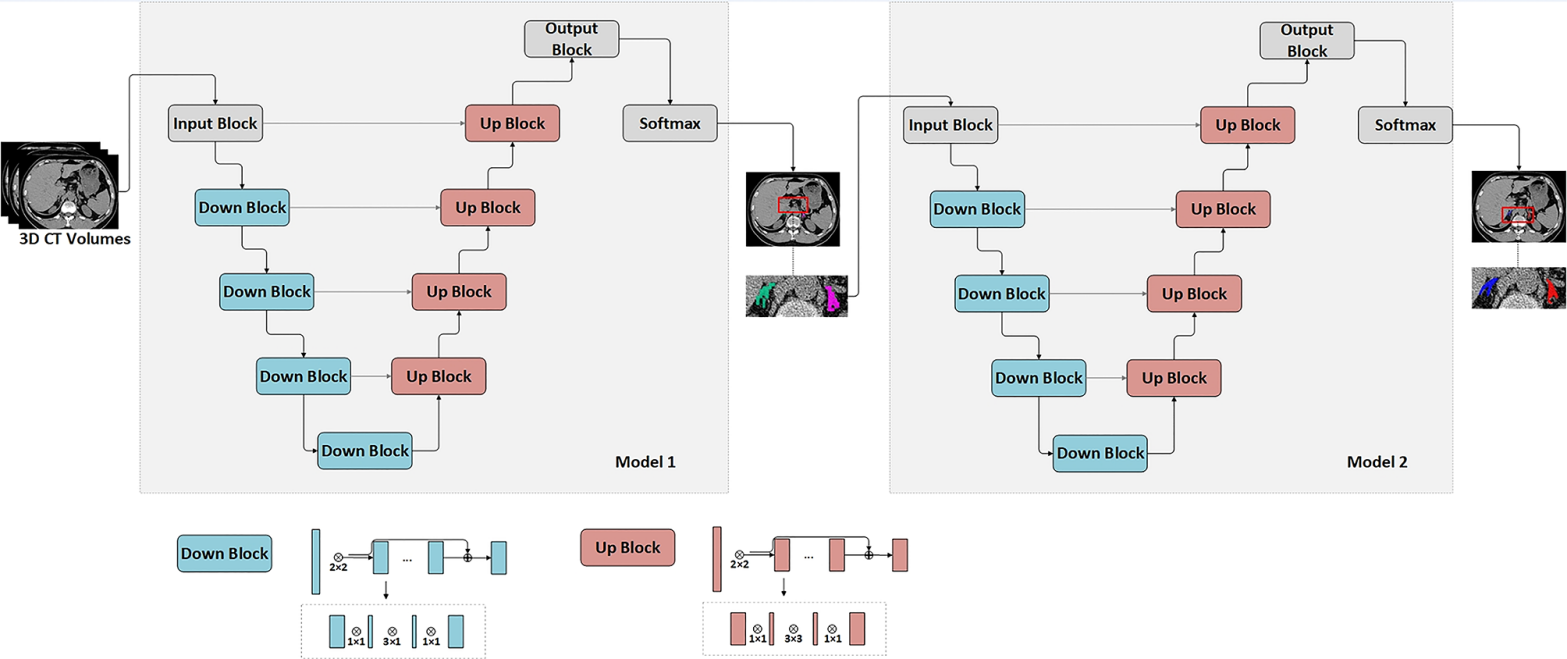
The framework of the used V-net

We proposed a cascaded V-net network framework to automatically segment the bilateral adrenal glands. The cascaded V-net network framework was constructed using two V-nets based on the coarse-to-fine principle, including a coarse segmentation network for rapidly locating the target area, namely the adrenal glands, and an exemplary segmentation network for optimization and precisely delineating the adrenal glands. Coarse segmentation adopts 3 × 3 × 3 resolution to quickly find the target region (global sampling) from the global, and fine segmentation adopts 1 × 1 × 1 to carry out more detailed segmentation (mask sampling) in the target region. Dice loss is used as a loss function. ROI of periadrenal fat was obtained using the inflation algorithm based on the adrenal gland. An experienced radiologist (Y.F.) manually delineated the ROIs of bilateral adrenal glands, which werethe ground truth labels of adrenal, and he was asked to delineate the adrenals according to the image delineating principles of BraTS 2018. They compared the automated segmentation with the ground truth; any discrepancies or errors identified during the review are manually corrected or adjusted (14–15). We trained a coarse localization model as Model 1 for the auto-segmentation framework, which can perform coarse segmentation to locate the adrenal gland area. The second V-net model was used to define segmentation as Model 2 and further divided into the left and right adrenal glands. The segmentation frame is shown in Fig. 2. ROIs, including bilateral adrenal glands and periadrenal fat from all CT images without annotation, were segmented using the auto-segmentation framework. Dice value was used to evaluate the cascaded V-net network framework.

### Radiomics feature selection and models building

Radiomic features were extracted from ROIs on the CT images using a Python package (PyRadiomics V3.0). B-spline interpolation resampling was used to normalize the voxel size, and the anisotropic voxels were resampled to form isotropic voxels of 1.0 mm × 1.0 mm × 1.0 mm in the feature extraction. A total of 2,264 radiomics features of each ROI were extracted. Next, the feature in the training set was preprocessed by standardization. The mean and variance in the training set were applied to the validation and test sets.

First, a univariate analysis named K-best was employed to reduce dimensionality and select significant features. It selected features according to K’s highest scores computed through ANOVA F-value and P-value between label and features. Features with a significant difference (*P* < 0.05) were selected. Next, the LASSO feature-selection algorithm extracted the most informative radiomics features to prevent the “curse of dimensionality.“ The LASSO algorithm assigns a penalty (lambda) to each feature based on its contribution to the model’s performance. The optimal regularization parameter (lambda) for the LASSO algorithm is typically determined using 10-fold cross-validation. By varying the value of lambda, only the most essential features that contribute significantly to predicting the outcome are included in the final radiomics model. After feature extraction and selection, logistic regression (LR) algorithms were trained to construct three radiomics models (AM, PM, and FM) for predicting the disease progression of COVID-19. We used five-fold cross-validation to find the optimal hyperparameters of the logistic regression model, then used the optimal hyperparameters to construct models. Lbfgs solver was used in the optimization problem. In the FM model, the RadScore of the patient was calculated according to the LASSO algorithm.

### RN construction and evaluation

We used univariate analysis to assess the relationship between clinical factors plus serum biomarkers and disease outcome. Features with P < 0.05 were introduced into multivariate LR analysis, and the optimal radiomics feature subset was selected by LASSO five-fold cross-validation. The two make the best combination. The best training and validation sets assignment was chosen for the subsequent analysis. Next, we applied the multivariate LR model to build CM using valuable clinical indicators and RN using the RadScore from FM with clinical indicators to predict the disease progression of COVID-19.

We diagnosed collinearity by calculating the VIF for variables in RN to detect multicollinearity among the radiomics nomogram variables. In the end, RN was verified in the validation and test sets. Calibration curves and the Hosmer–Lemeshow test assessed the relation between the predicted risks and actual results. DCA was used to evaluate the performance of the RN.

### Statistics

Before model building, differences in clinical factors and serum biomarkers between moderate and severe patient sets were assessed using the Mann-Whitney U test or Student’s *t*-test for continuous variables and the χ2 test or Fisher’s exact test for categorical variables. We analyzed all data using SPSS for Windows version 26.0 (IBM Corp., Armonk, New York, USA). *P* < 0.05 was considered a statistically significant difference.

Dice value was used to assess the effectiveness of the auto-segmentation framework. The AUC of receiver-operating characteristics (ROC) with 95% confidence interval (95% CI), sensitivity, and specificity were used to evaluate the performance of AM, PM, FM, CM, and RN. Accuracy was calculated to assess the prediction performance. Differences in AUC values among different models were estimated using the DeLong test. Calculate three statistics for each pair of models being compared: AUC - ROC, variance of AUC - ROC, and covariance of AUC - ROC. The DeLong test whether there is a significant difference between the AUC - ROC values of the two models based on their variances and covariance.

## Results

### Patient characteristics

The data of 1,209 patients were formed as our training and validation sets, including 672 patients from moderate COVID-19 and 537 from severe COVID-19. The patient characteristics in training and validation sets are listed in Table 1. No significant differences were observed between the training and validation set in sex (*P* = 0.238). IL-6, WBC, L, N, CRP, PCT, PT, DD, BS, AST, ALB, D-Bil, BNP, LDH, and CK-MB differed significantly between moderate and severe pneumonia sets both in training and validation sets (*P* < 0.05).

**Table 1:** Clinical characteristics of patients in training and validation set (n = 1209)

Variable		Training set				Validation set			
	Moderate pneumonia	Severe pneumonia	*P*		Moderate pneumonia	Severe pneumonia		*P*	*P*
Age (yr,mean ± SD )	57.251 ± 13.660	63.121 ± 12.390	0.000		58.396 ± 12.299	65.667 ± 12.195		0.000	0.011
Sex n(%)			0.476					0.069	0.238
							^		
Men	270(50.2%)	226(52.7%)			64(47.8%)	65(60.2%)			
women	268(49.8%)	203(47.3%)			70(52.2%)	43(39.8%)			
IL-6	3.164 ± 4.940	16.792 ± 100.663	0.000		2.613 ± 2.513	7.180 ± 16.006		0.000	0.003
WBC(109/L)	5.966 ± 1.886	6.384 ± 2.476	0.000		5.647 ± 1.941	6.572 ± 2.609		0.003	0.243
L(10^9^/L)	1.646 ± 0.601	1.423 ± 0.680	0.000		1.434 ± 0.520	1.150 ± 0.568		0.000	0.000
N(109/L)	3.703 ± 1.638	4.320 ± 2.388	0.000		3.617 ± 1.781	4.852 ± 2.564		0.000	0.323
HB(g/L)	125.507 ± 17.062	117.773 ± 23.603	0.000		122.284 ± 14.681	117.082 ± 23.804		0.096	0.021
RBC(1012/L)	4.066 ± 0.547	3.869 ± 0.605	0.000		3.948 ± 0.497	3.876 ± 0.559		0.208	0.043
PLT(10^9^/L)	231.213 ± 72.781	231.301 ± 85.677	0.727		265.530 ± 88.988	232.269 ± 78.348		0.012	0.001
CRP(µg/L)	3.211 ± 3.393	5.002 ± 4.483	0.000		5.008 ± 3.962	7.478 ± 3.727		0.000	0.000
PCT(µg/L)	1.877 ± 2.647	1.498 ± 2.434	0.003		1.396 ± 2.420	1.242 ± 2.247		0.020	0.051
PT(s)	11.778 ± 2.825	12.596 ± 4.826	0.000		12.236 ± 2.154	12.547 ± 3.287		0.042	0.100
TT(s)	13.549 ± 3.623	14.692 ± 5.088	0.000		14.974 ± 3.236	15.086 ± 3.892		0.071	0.000
FIB (g/L)	3.408 ± 1.095	3.398 ± 0.941	0.097		3.373 ± 0.925	3.768 ± 1.010		0.000	0.002
DD (mg/L)	1.256 ± 1.937	1.720 ± 2.516	0.000		1.479 ± 2.038	3.249 ± 4.969		0.000	0.000
BS (mmol/L)	5.487 ± 2.123	6.031 ± 2.747	0.000		5.295 ± 1.660	6.053 ± 2.656		0.005	0.880
ALT (u/L)	32.599 ± 35.669	33.361 ± 35.745	0.563		30.765 ± 23.527	37.676 ± 30.337		0.087	0.039
AST (u/L)	22.623 ± 13.871	25.920 ± 19.039	0.008		24.469 ± 17.138	33.966 ± 45.357		0.009	0.007
TP (g/L)	65.851 ± 7.689	63.932 ± 8.994	0.000		62.295 ± 8.667	61.614 ± 6.204		0.059	0.000
ALB (g/L)	38.369 ± 4.747	36.289 ± 5.404	0.000		35.805 ± 5.214	33.883 ± 3.949		0.000	0.000
TBil (µmol/L)	10.544 ± 6.458	10.597 ± 5.935	0.881		9.417 ± 4.131	12.193 ± 7.718		0.000	0.718
D-Bil (µmol/L)	3.734 ± 3.974	4.063 ± 3.767	0.028		3.494 ± 1.945	5.095 ± 4.819		0.000	0.083
BUN (mmol/L)	4.672 ± 1.493	5.168 ± 2.408	0.006		4.471 ± 3.139	4.881 ± 2.415		0.143	0.000
Cre (µmol/L)	66.616 ± 17.565	68.839 ± 27.524	0.938		71.733 ± 54.602	70.131 ± 23.705		0.578	0.913
BNP (pg/ml)	14.952 ± 63.098	40.715 ± 116.651	0.000		14.357 ± 38.344	49.172 ± 90.294		0.000	0.174
Mb(µg/L)	6.673 ± 5.176	11.440 ± 35.630	0.104		8.234 ± 19.045	34.481 ± 219.515		0.000	0.956
Tn(µg/L)	3.279 ± 2.815	2.879 ± 2.844	0.278		3.493 ± 2.782	2.696 ± 2.870		0.057	0.413
LDH (u/L)	172.169 ± 58.910	213.429 ± 87.814	0.000		193.202 ± 58.210	269.167 ± 131.487		0.000	0.000
CK (u/L)	57.192 ± 41.249	58.817 ± 75.964	0.003		55.940 ± 68.155	85.376 ± 133.713		0.364	0.423
CK-MB (u/L)	9.233 ± 6.598	10.957 ± 9.737	0.001		8.793 ± 4.359	10.668 ± 8.066		0.030	0.905

Clinical characteristics and serum biomarkers of patients in the training and internal validation set. IL-6 = Interleukin 6; WBC = white blood cell count, L = lymphocyte count, N = neutrophil count, HB = hemoglobin; RBC = red blood cell; PLT = blood platelet count; CRP = C reactive protein; PCT = procalcitonin; PT = prothrombin Time; TT = thrombin time; FIB = fibrinogen; DD = D dimer; BS = blood sugar; ALT = alanine transaminase; AST = glutamic oxaloacetic transaminase; TP = total protein; ALB = albumin; TBil = total bilirubin; D-Bil = direct bilirubin; BUN = blood urea nitrogen; Cre = creatinine; BNP = B-type natriuretic peptide; Mb = myoglobin; Tn = troponin; LDH = lactate dehydrogenase; CK = creatine kinase; CK-MB = creatine kinase-MB

Note: P-value < 0.05 was considered as a significant difference. Differences in clinical factors and serum biomarkers between moderate and severe patient sets were assessed using the Mann–Whitney U test or Student’s t-test for continuous variables depending on the standard test and the χ2 test or Fisher’s exact test for categorical variables

### Adrenal gland and periadrenal fat auto-segmentation framework

**Fig. 3:**
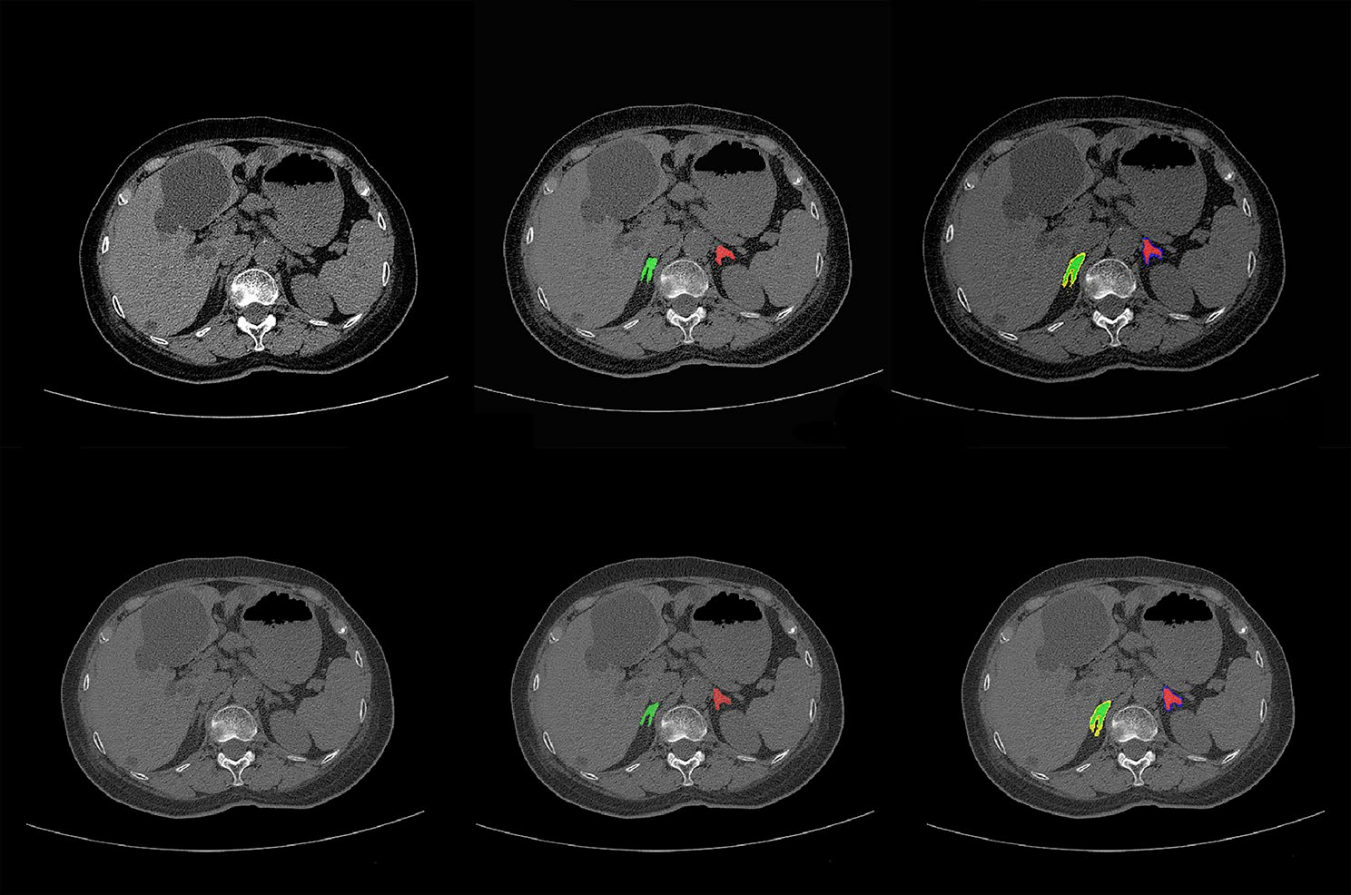
The comparison of auto-segmentation results and ground truth on representative cases from the training set. The upper line shows the ground truth, and the lower line shows the auto-segmentation results. The green color shows the right adrenal glands, whereas red denotes the left. Yellow shows right periadrenal fat, and purple shows left

For adrenal gland segmentation, we manually delineated bilateral adrenal glands from the CT images of 315 patients; 265 were used for training. The remaining data from 50 patients were used to evaluate the performance. The segmentation model yielded average Dice values of 79.48% for the left and 78.55% for the right adrenal gland. The entire adrenal gland achieved an average Dice value of 79.02%. Representative auto**-**segmentation results are shown in Fig. 3. The segmentation algorithm was then used to segment all the remaining data automatically.

### Radiomics feature and clinical indicator selection

In the training set, the number of radiomics features was reduced to 23 for building AM that included 8 first-order features and 15 texture features (Gray Level Co-occurrence Matrix [GLCM] = 3, Gray Level Size Zone Matrix [GLSZM] = 8, Gray Level Run Length Matrix [GLRLM] = 2 and Gray Level Dependence Matrix [GLDM] = 2); 68 for PM that included 11 first-order features, 2 sharp feature, and 55 texture features (GLCM = 13, GLSZM = 25, GLRLM = 7, GLDM = 9 and Neighboring Gray Tone Difference Matrix [NGTDM] = 1) and 82 for FM that included 12 first-order features and 70 texture features (GLCM = 17, GLSZM = 38, GLRLM = 3, GLDM = 5 and NGTDM = 7). These features were evaluated to construct three radiomics models.

**Fig. 4:**
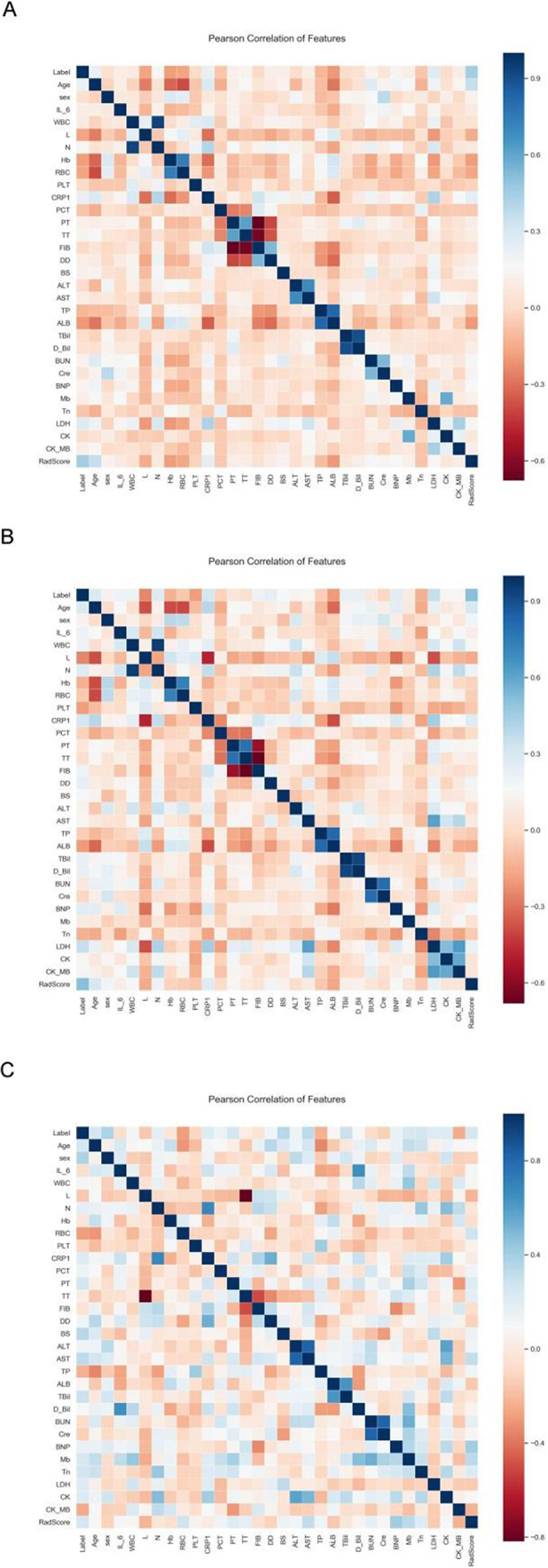
Correlation heatmap in training, validation, and test set. FM = fusion of adrenal gland and periadrenal fat model; CM = clinical model; RN = radiomics nomogram

A total of 30 clinical factors and serum biomarkers were analyzed in our study. Next, univariate logistic regression analysis selected 17 clinical factors and serum biomarkers. They are LDH, CRP, age, ALB, L, N, Hb, RBC, DD, BS, WBC, CK_MB, TT, BUN, AST, TP, PT. and 7 indicators, LDH, L, HB, DD, WBC, TT, and TP, were selected using multivariate logistic regression analysis. The relationship between RadScore from FM used in constructing a radiomics nomogram (RN) and 30 clinical factors plus serum biomarkers were analyzed using Pearson correlation between training, validation, and test sets (Fig. 4). The difference in RadScores with clinical factors or serum biomarkers was not significant. Then, 17 clinical factors and serum biomarkers were selected using univariate logistic regression analysis, and 7 indicators, LDH, L, HB, DD, WBC, TT, and TP, were selected using multivariate logistic regression analysis.

### Three radiomics models and clinical model building

**Fig. 5:**
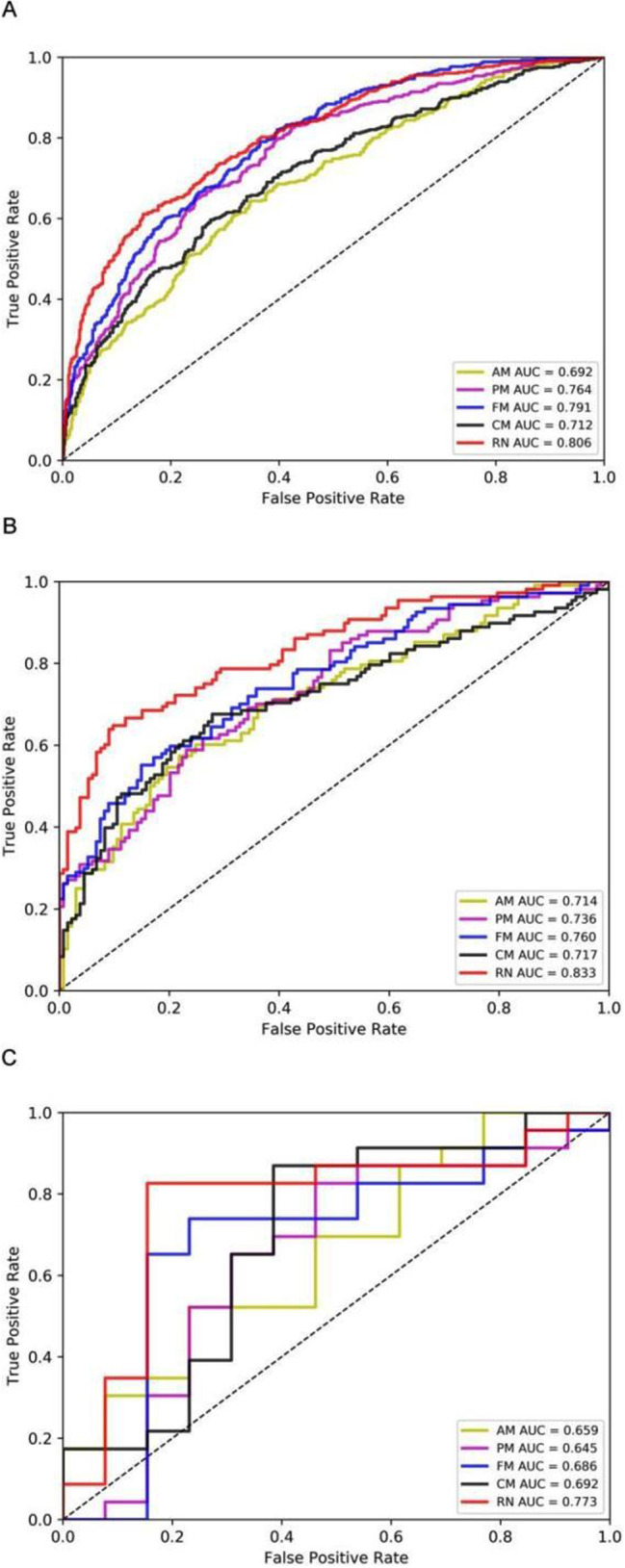
ROC curves in training, validation, and test sets. AM = adrenal gland model; PM = periadrenal fat model; FM = fusion of adrenal gland and periadrenal fat model; CM = clinical model; RN = radiomics nomogram

We developed three radiomics models (AM, PM, FM) based on radiomics features and a clinical model (CM) based on the seven selected independent predictive clinical indicators. We used three evaluation indicators (area under the curve [AUC], 95% CI, sensitivity [SEN], and specificity [SPE]) to assess AM, PM, FM, and CM for predicting the progression of patients with COVID-19 in training, validation, and test sets. In general, AM achieved an AUC of 0.692, 0.714, and 0.659 in the training set, validation set, and test set, respectively; PM achieved an AUC of 0.764, 0.736, and 0.645; FM achieved an AUC of 0.791, 0.760 and 0.686; CM obtained an AUC of 0.712,0.717 and 0.692 (Fig. 5, supplementary Table 2).

**Fig. 6:**
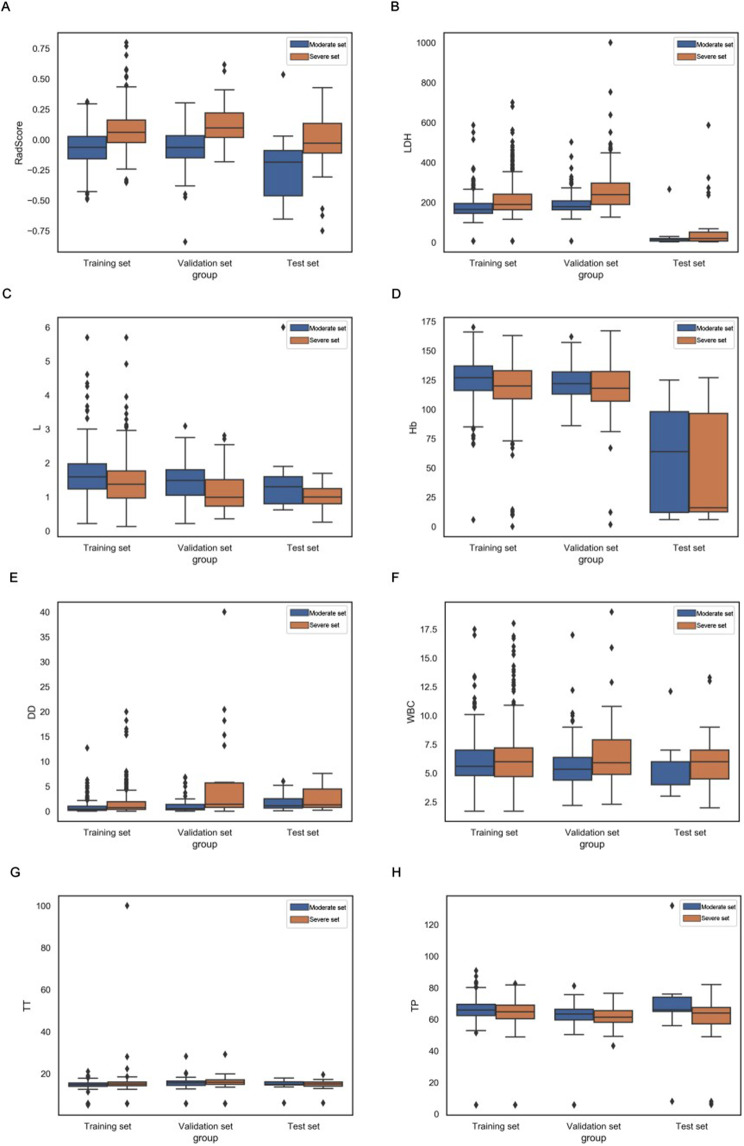
Box plots summarizing rad score and 7 clinical indicators between moderate and severe patient sets in training, validation, and test sets. In each box plot, the horizontal line crossing the box is the median, and the bottom and top are the lower and upper quartiles. FM = fusion of adrenal gland and periadrenal fat model; CM = clinical model; RN = radiomics nomogram; LDH = lactate dehydrogenase; L = lymphocyte count; Hb = hemoglobin, DD = D dimer; WBC = white blood cells count; TT = thrombin time and TP = total protein

Box plots summarizing the RadScores and coefficients of seven clinical indicators in training, validation, and test sets directly demonstrate the difference between RadScore and coefficients of seven clinical indicators between the moderate and severe patient sets (Fig. 6).

### RN construction and validation

**Fig. 7:**
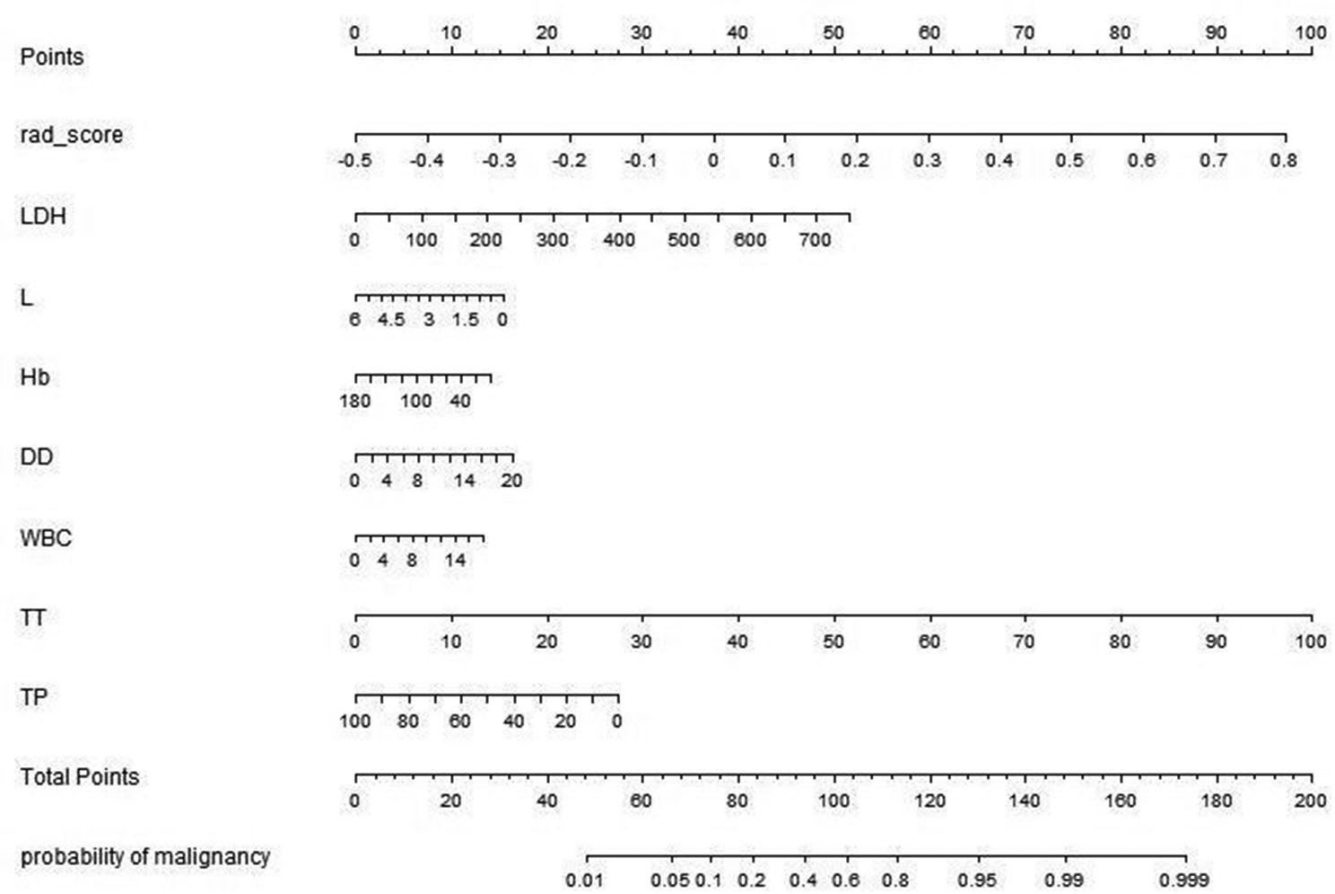
Radiomics nomogram developed in training set with radiomics features, lactate dehydrogenase (LDH), lymphocyte count (L), hemoglobin (Hb), D dimer (DD), white blood cells count (WBC), thrombin time (TT) and total protein(TP). Points are assigned for each variable by drawing a line upward from the corresponding variable to the Points line. The sum of points plotted on the total Points line corresponds with the severity of patients with COVID-19.

Multivariate analysis revealed that RadScore and seven clinical indicators were significant independent factors predicting disease progression in patients with COVID-19. We conducted collinearity diagnosis by calculating the VIF for variables in RN to detect multicollinearity among the radiomics nomogram variables, and the threshold was set to 10 [16–18]. Finally, the VIF value for the radiomics score and seven clinical indicators in RN ranged from 1.007 to 1.191, indicating no severe collinearity in these factors. Next, we used the RadScore from FM combined with seven clinical indicators to construct the RN to assess disease progression in patients with COVID-19 (Fig. 7). The RN showed satisfactory performance in predicting and assessing progression in patients with COVID-19 with an AUC of 0.806 (95% CI, 0.780 to 0.831) in the training set, 0.833 (95% CI, 0.780 to 0.878) in the validation set, and 0.773 (95% CI, 0.603 to 0.895) in the test set (Fig. 5, supplementary Table 2).

DeLong’s test was used to compare the AUCs of the training set’s three radiomics models, CM and RN. The result showed that the RN and FM were significantly better than CM (*P* < 0.0001). The difference between FM and RN was not statistically significant (*P* = 0.233) in the validation and test sets.

**Fig. 8:**
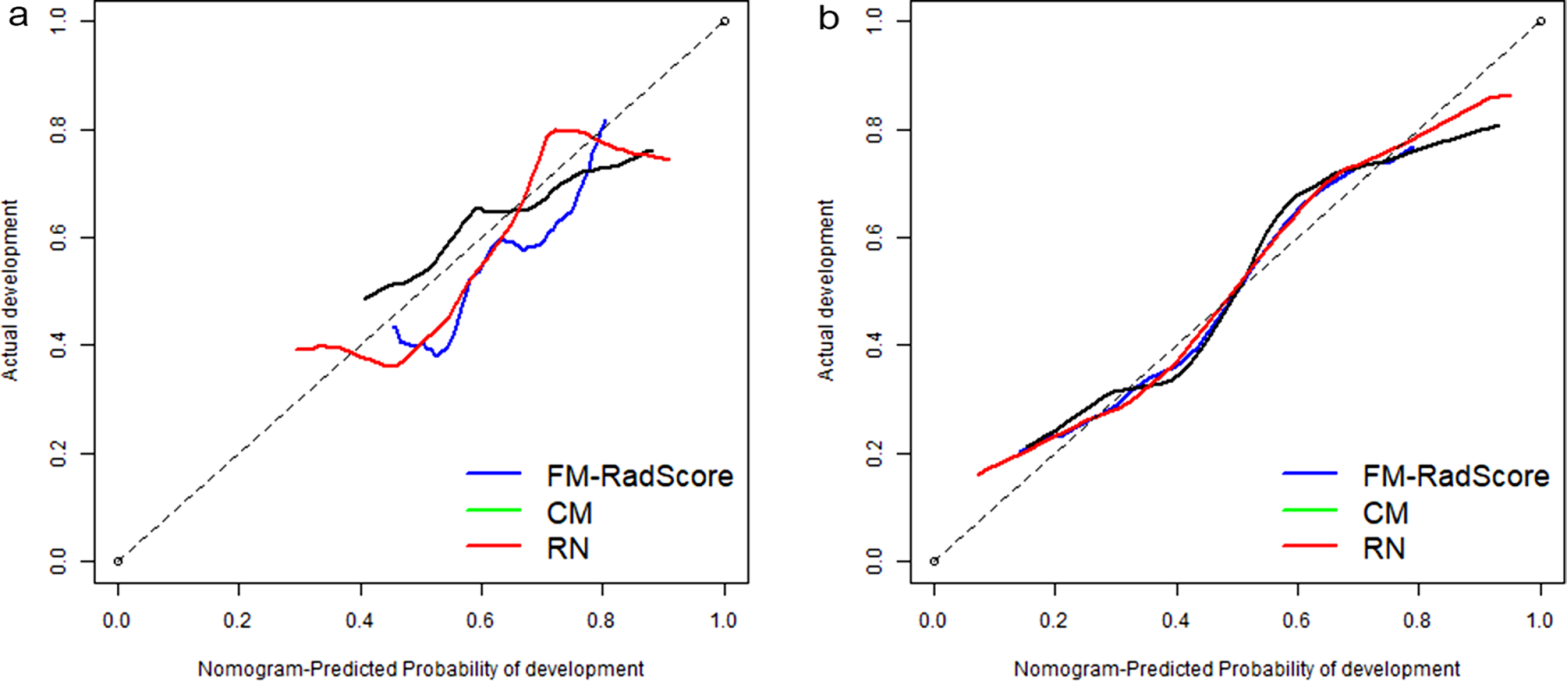
Calibration curves of the radiomics nomogram for predicting the disease progression of COVID-19 pneumonia in validation set (A) and test set (B). The y-axis represents the probability of COVID-19 pneumonia becoming severe, and the x-axis represents the predicted risk. The dashed line was the reference line where an ideal nomogram would lie. The dotted line was the performance of the radiomics nomogram, while the solid line corrected for any bias in the radiomics nomogram. MAE is 0.04 in the validation set and 0.075 in the test set

**Fig. 9:**
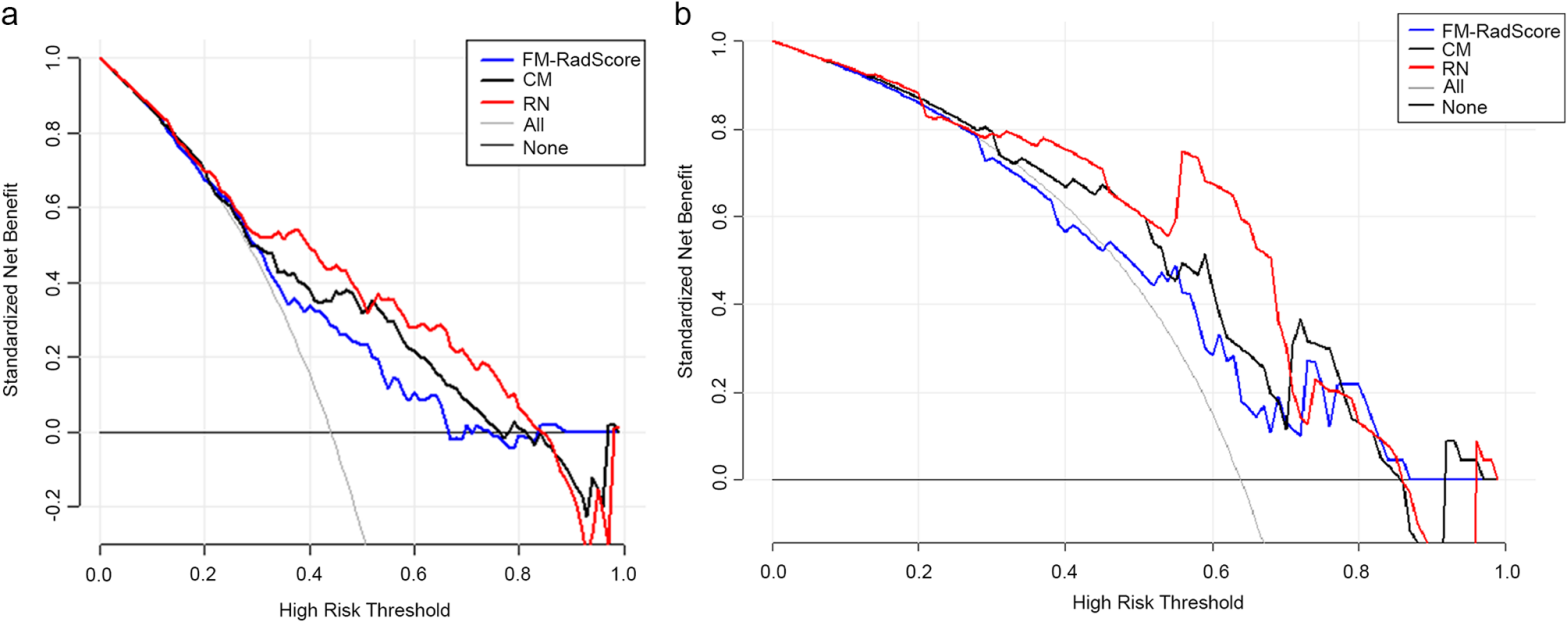
Decision curve analysis (DCA) for the radiomic model, clinical model, and radiomics nomogram. The y-axis measures the net benefit. Using the clinical model, radiomic model, and radiomics nomogram in the study to predict COVID-19 pneumonia progress adds more benefit than the treat all patients as severity patients scheme or the treat none scheme. The net benefit of the radiomics nomogram was better than clinical and radiomic models in both sets, with several overlaps in the training set

The Hosmer–Lemeshow test was not significant in the validation set (mean absolute error [MAE] = 0.075) or test set (MAE = 0.04), which suggests that there was no significant departure from actual values (Fig. 8). Decision curve analysis (DCA) (Fig. 9) showed that if the threshold probability was between 0.4 and 0.8 in the validation set, the RN could get more net benefits than FM and CM. If the threshold probability was between 0.3 and 0.7 in the test set, RN can still get more net benefits than FM and CM. The threshold cannot be set above 0.8; otherwise, the net benefit would become negative values.

## Discussion

In our study, the auto-segmentation framework could robustly localize the adrenal glands and accurately refine their boundary. The RN using adrenal glands and periadrenal fat onset CT images performed well, reflecting that the microscopic changes in adrenal glands and periadrenal fat in patients with COVID-19 can be detected using radiomics features. We hope this model will assist radiologists and clinicians in early intervention, monitoring, and management, ultimately leading to improved clinical outcomes. It can be used in the COVID-19 epidemic, especially when there is a shortage of healthcare workers.

Our results suggested that adrenal gland and periadrenal fat changes on the onset of CT images in severe patients differed from those in moderate patients. Results from autopsies in 10 patients from COVID-19 performed by Zinserling et al. [19] showed that inflammation—small proliferations of cells with enlarged light nuclei and mononuclear infiltration, CD3 + and CD8 + in different layers of adrenal glands and their surrounding tissue, such as periadrenal fat. These changes may be related to direct damage of adrenal glands and indirect changes stimulated by systemic inflammation caused by SARS-CoV-2 or the immune state. In one respect, the changes were caused by direct damage done by SARS-CoV-2. The SARS virus has been identified in adrenal cells, suggesting a direct local replication-mediated cytopathic effect of the virus in adrenal tissue [20]. Moreover, the virus may cause hemorrhage, necrosis, or thrombosis at the adrenal level [21]. Adrenal lesions due to influenza and other viruses have been described previously [22]. Recent findings have indicated the possibility of venous thromboembolism in patients with COVID-19, which may cause an acute adrenal insufficiency that may be an indicator of making the disease worse [23]. All of these changes caused by the COVID-19 virus can change the physical properties of the adrenal tissue and the heterogeneity between tissues and cells in patients with different conditions. First-order and higher-order radiomics features can reflect the changes in physical properties. We selected 82 features, concluding with 12 first-order features and 70 texture features. In FM, the most relevant to the severity of the disease is Robust - Mean - Absolute - Deviation and there is a negative correlation. This feature reflects the amount of variation or dispersion from the mean value which means the higher the feature is, the less severe the disease is. Moreover, in the AM model, we got the same feature with the highest negative correlation with disease severity. The most relevant positive correlation feature is Zone Entropy which reflects the uncertainty or randomness in the distribution of zone sizes and gray levels. A higher value indicates more heterogeneneity in the texture patterns which means more severity of disease. In PM, The feature with the largest positive correlation is also Zone Entropy. This feature reflects the heterogeneity of adrenal glands, which is the same as that extracted feature by tumor radiomics analysis [24]. Our predicting results showed that AM’s AUC value was slightly better than PM’s, but there was no statistical significance. That may indicate that the inner changes in adrenal glands were not different from the changes in periadrenal fat. Further research is needed to confirm whether changes in periadrenal fat were caused by adrenal gland inflammatory cells’ infiltration or direct damage of SARS-CoV-2 [25].

Previous research also reported that rather than direct damage by SARS-CoV-2 itself, adrenal gland changes were believed to be caused by immune overreaction or cytokine storm-inducing endocrinological pathway impaired through a tight binding mechanism of SARS-CoV-2 and ACE2 [26]. Endocrinological pathway impairment may be related to the following aspects. First, ACE2 is highly expressed in human adrenals, which serves as the entry receptor for SARS-CoV-2 that is bound to be significantly affected. Some work has proposed that the imbalanced ACE/ACE2 axis may mediate lung tissue repair and wound healing pathways [27]. Second, the primary substrate for ACE2 is angiotensin II. ACE2 is a negative regulator of the renin-angiotensin-aldosterone system (RAAS) by converting the active angiotensin and angiotensin II to the inactive angiotensin 1–7 [28]. Finally, the SARS virus contains several permutations of amino acid sequences with homology to the antigenic relevant residues of ACTH [29] that will have potential and significant pathophysiological effects due to molecular mimicry. Potential cross-reactivity of antibodies may cause local infiltration with immunocompetent cells in adrenal glands [19].

In summary, these aspects suggested that adrenal cells and surrounding tissue damage in patients with COVID-19 may be caused by a viral infection and further secondary inflammatory plus autoimmune processes located in the adrenal glands. Due to the pandemic of COVID-19, scholars have done much research on computer modeling of COVID-19. Many diagnostic models are machine learning models based on chest CT for detecting COVID-19 infection and predictive models for predicting the risk of death, progression of severe disease, or the length of hospital stay (30). Among COVID-19 patients, the most common predictors of severe prognosis include age, sex and radiomics features, C-reactive protein, lactate dehydrogenase, and lymphocyte counts. These results are consistent with the univariate logistic regression analysis (31). There was little evaluation of the calibration of the predictions, and many prediction models are mainly based on clinical data (32). Compared with other models, we adopted the adrenal grand CT images as the study’s data, which could be used in multi-organ fusion prediction in the future.

## Conclusions

We automatically obtained the ROI from the onset of CT images through the auto-segmentation framework and inflation algorithm. We proposed and constructed an adrenal gland auto-segmentation framework based on chest CT images and AI technology. Until now, we have not found any research using adrenal gland CT parameters as indicators to evaluate the progression in patients with COVID-19, especially in the radiomics field. Our work has some limitations in the data collection process. First, we used the chest CT images as data resources, which would affect the visualization and delineation of adrenal tissue. However, considering clinical practicality and radiation to patients, there is no need to perform another CT scan using professional adrenal glands CT parameters to observe adrenal lesions better because CT examination of patients with COVID-19 is mainly to detect and observe pulmonary lesions. Second, unlike other studies, we selected the entire organ as ROIs rather than the lesion. The periadrenal fat area may not be precise and may contain some other indistinguishable tissue from the human eye.

### Electronic supplementary material

Below is the link to the electronic supplementary material.

Supplementary Material 1

## Data Availability

The data used and/or analyzed during the current study are available from the corresponding author on reasonable request. The datasets generated during and/or analyzed during the current study are available from the corresponding author on reasonable request. The code used and/or analyzed during the current study are available from the corresponding author on reasonable request.

## List of abbreviations

LASSO: Least absolute shrinkage and selection operator
CM: Clinical model AM:adrenal gland model
PM: Periadrenal fat model
FM: Fusion of adrenal gland and periadrenal fat model
RN: Radiomics nomogram model
ROI: Region of interest
MAE: Mean absolute error
PCR: Polymerase chain reaction
AI: Artificial intelligence
AUC: Under the curve
ACTH: Adrenocorticotropic hormone
SNS: Sympathetic nervous system
ACE2: Angiotensin-converting enzyme-2
HSH: Huoshenshan Hospital
MCH: Maternal and Child Health Hospital Optical Valley Branch Hospital
TTH: Taikang Tongji (Wuhan) Hospital
GLCM: Gray-level co-occurrence matrix
GLRLM: Gray-level run-length matrix
GLSZM: Gray-level size zone matrix
GLDM: Gray-level dependence matrix
NGTDM: Neighboring Gray Tone Difference Matrix
LR: Logistic regression
DCA: Decision curve analysis
IL-6: Interleukin 6
L: Lymphocyte count
CRP: C-reactive protein
PCT: Procalcitonin
DD: D dimer
AST: Glutamic oxaloacetic transaminase
BNP: B-type natriuretic peptide
LDH: Lactate dehydrogenase
CK: Creatine kinase
CK-MB: Creatine kinase-MB
WBC: White blood cell count
N: Neutrophil count
HB: Hemoglobin
RBC: Red blood cell count
PLT: Blood platelet count
PT: Prothrombin time
TT: Thrombin time
FIB: Fibrinogen
BS: Blood sugar
ALT: Alanine transaminase
TP: Total protein
ALB: Albumin
TBil: Total bilirubin
D-Bil: Direct bilirubin
BUN: Blood urea nitrogen
Cre: Creatinine
Mb: Myoglobin
Tn: Troponin
CK: Creatine kinase
SEN: Sensitivity
SPE: Specificity
VIF: Variance inflation factor
RAAS: Renin-angiotensin-aldosterone system
