# Supplementary material for: A radiomics based approach using adrenal gland and periadrenal fat CT images to allocate COVID-19 health care resources fairly
Source: BMC Med Imaging. 2023 Nov 10;23:181. doi: 10.1186/s12880-023-01145-9 (PMC10636917; doi:10.1186/s12880-023-01145-9)
Supplement: Supplementary file 1 — Supplementary Material 1 [file 12880_2023_1145_MOESM1_ESM.docx]

Supplementary figures and tables

Supplementary table 1. CT characteristics

| Manufacture | Scanner type | Rotation time | Resolution | Tube voltage (kv) | Tube current (mAs) | Section thickness | Reconstruction  technique | Algorithm |
| --- | --- | --- | --- | --- | --- | --- | --- | --- |
| SIEMENS | SOMATOM  Perspective | 0.6s | 512x512 | 110 | 49 | 1 | ADMIRE 3 | I50s |
| SIEMENS | SOMATOM  Definition | 0.5s | 512x512 | 100 | 100 | 1 | FBP | B50f medium sharp |
| SIEMENS | SOMATOM  Definition AS+ | 0.5s | 512x512 | 120 | 52 | 0.6 | FBP | B50f medium sharp |
| SIEMENS | SOMATOM Force | 0.5s | 512x512 | 100 | 52 | 1 | ADMIRE 3 | BI57 |
| GE MEDICAL  SYSTEMS | BrightSpeed | 0.5s | 512x512 | 100-120 | 120-140 | 1.25 | FBP | chest,lung,stnd |
| GE MEDICAL  SYSTEMS | LightSpeed VCT | 0.5s | 512x512 | 100-120 | 120-140 | 0.625 | FBP | chest,lung,stnd |
| NMS | NeuViz 16  Essence | 0.5s | 512x512 | 120 | 100-120 | 1.25 | Iterate | lung |
| UIH | uCT510 | 0.5s | 512x512 | 120 | 120-150 | 1.5 | FBO | KARL 3D®iterative |

Manufacture: machine manufacturer's brand; Scanner type: CT scanner type; Rotation time: the time required for the CT machine to complete one full rotation; Resolution: the smallest object size that the CT scanner can distinguish; Tube voltage: the voltage applied to the X-ray tube in a CT scanner; Tube current: the amount of current flowing through the X-ray tube during a CT scan; Section thickness: the thickness of the individual image slices; Reconstruction technique: process of creating cross-sectional images; Algorithm: technique for the reconstruction of CT images.

Supplementary Table 2. Predictive performances of models in training, validation and test set.

Variable Training set Validation set Test set

|  | AUC(95%CI) | SEN | SPE | AUC(95%CI) | SEN | SPE |  | AUC(95%CI) | SEN | SPE |
| --- | --- | --- | --- | --- | --- | --- | --- | --- | --- | --- |
| AM | 0.692(0.662-0.721) | 0.643 | 0.652 | 0.716(0.655-0.772) | 0.638 | 0.656 |  | 0.659(0.482-0.808) | 0.696 | 0.539 |
| PM | 0.763(0.735-0.789) | 0.651 | 0.749 | 0.736(0.675-0.790) | 0.654 | 0.664 |  | 0.645(0.469-0.797) | 0.652 | 0.692 |
| FM | 0.791(0.763-0.816) | 0.672 | 0.740 | 0.760(0.701-0.813) | 0.682 | 0.681 |  | 0.686(0.510-0.830) | 0.696 | 0.769 |
| CM | 0.712(0.682-0.740) | 0.613 | 0.700 | 0.717(0.655-0.772) | 0.676 | 0.724 |  | 0.692(0.517-0.835) | 0.872 | 0.615 |
| RN 0.806(0.780-0.831) | | 0.657 | 0.775 | 0.833(0.780-0.878) | 0.722 | 0.791 | 0.773(0.603-0.895) | | 0.826 | 0.846 |

ACC = accuracy; SEN = sensitivity; SPE = specificity; AM = adrenal gland model; PM

= periadrenal fat model; FM = fusion of adrenal gland and periadrenal fat model; CM = clinical model; RN = radiomics nomogram

Supplementary Table 3. The extracted features

| Category (Quantity) | Radiomic Features |
| --- | --- |
| Filter (n_f=14) | BoxMean, AdditiveGaussinNoise, BinomialBlurImage, CurvatureFlow, BoxsigmaImage, LoG, Wavelet, Normalize, LaplacianSharpening, DiscreteGaussian, Mean, SpeckleNoise, RecursiveGaussian, ShotNoise |
| Shape-based (n=14) | Area, Perimeter, Sphericity, Elongation, Extent, Circularity, Solidity, Eccentricity, Equivalent diameter, Major axislength, Minor axis length, Perimeter to area ratio, Maximum 2D diameter, Spherical disproportion |
| First Order Statistics (n=450) | Energy, Total Energy, Entropy, Minimum, 10th percentile, 90th percentile, Maximum, Mean, Median, Interquartile Range, Range, Mean Absolute Deviation (MAD), Robust Mean Absolute Deviation (rMAD), Root Mean Squared (RMS), Standard Deviation, Skewness, Kurtosis, Variance, Uniformity |
| Gray Level Cooccurence Matrix (GLCM) (n=525) | Autocorrelation, Joint Average, Cluster Prominence, Cluster Shade, Cluster Tendency, Contrast, Correlation, Difference Average, Difference Entropy, Difference Variance, Joint Energy, Joint Entropy, Informational Measure of Correlation (IMC)(1~2), Inverse Difference Moment (IDM), Maximal Correlation Coefficient (MCC), Inverse Difference Moment Normalized (IDMN), Inverse Difference (ID), Inverse Difference Normalized (IDN), Inverse Variance, Maximum Probability, Sum Average, Sum Entropy, Sum of Squares |
| Gray Level Run Length Matrix (GLRLM) (n=350) | Short Run Emphasis (SRE), Long Run Emphasis (LRE), Gray Level Non-Uniformity (GLN), Gray Level Non-Uniformity Normalized (GLNN), Run Length Non-Uniformity (RLN), Run Length Non-Uniformity Normalized (RLNN), Run Percentage (RP), Gray Level Variance (GLV), Run Variance (RV), Run Entropy (RE), Low Gray Level Run Emphasis (LGLRE), High Gray Level Run Emphasis (HGLRE), Short Run Low Gray Level Emphasis (SRLGLE), Short Run High Gray Level Emphasis (SRHGLE), Long Run Low Gray Level Emphasis (LRLGLE), Long Run High Gray Level Emphasis (LRHGLE) |
| Gray Level Size Zone Matrix (GLSZM) (n=400) | Small Area Emphasis (SAE), Large Area Emphasis (LAE), Gray Level Non-Uniformity (GLN), Gray Level Non-Uniformity Normalized (GLNN), Size-Zone Non-Uniformity (SZN), Size-Zone Non-Uniformity Normalized (SZNN), Zone Percentage (ZP), Gray Level Variance (GLV), Zone Variance (ZV), Zone Entropy (ZE), Low Gray Level Zone Emphasis (LGLZE), High Gray Level Zone Emphasis (HGLZE), Small Area Low Gray Level Emphasis (SALGLE), Small Area High Gray Level Emphasis (SAHGLE), Large Area Low Gray Level Emphasis (LALGLE), Large Area High Gray Level Emphasis (LAHGLE) |
| Neighbouring Gray Tone Difference Matrix (NGTDM) (n=400) | Coarseness, Contrast, Busyness, Complexity, Strength |
| Gray Level Dependence Matrix (GLDM) (n=125) | Small Dependence Emphasis (SDE), Large Dependence Emphasis (LDE), Gray Level Non-Uniformity (GLN), Dependence Non-Uniformity (DN), Dependence Non-Uniformity Normalized (DNN), Gray Level Variance (GLV), Dependence Variance (DV), Dependence Entropy (DE), Low Gray Level Emphasis (LGLE), High Gray Level Emphasis (HGLE), Small Dependence Low Gray Level Emphasis (SDLGLE), Small Dependence High Gray Level Emphasis (SDHGLE), Large Dependence Low Gray Level Emphasis (LDLGLE), Large Dependence High Gray Level Emphasis (LDHGLE) |


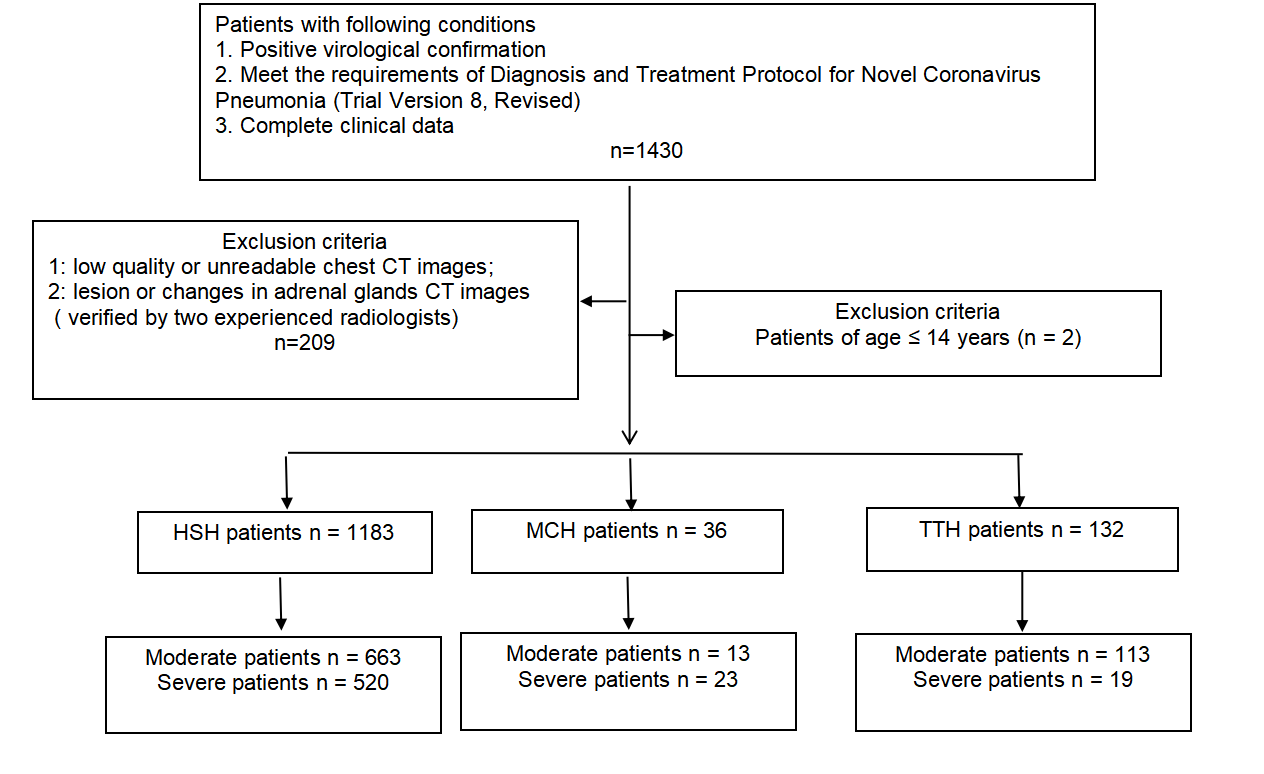


Supplementary figure 1. Flowchart shows the patient inclusion and exclusion criteria

Two radiologists based on clinical information, laboratory examination and CT results, evaluate possibilities of patients with adrenal lesions, there is the possibility, we excluded it.
